# Interactive Palliative and End-of-Life Care Modules for Pediatric Residents

**DOI:** 10.1155/2017/7568091

**Published:** 2017-02-12

**Authors:** Mindy K. Ross, Ami Doshi, London Carrasca, Patricia Pian, JoAnne Auger, Amira Baker, James A. Proudfoot, Mark S. Pian

**Affiliations:** ^1^Division of Pediatric Pulmonary and Sleep Medicine, University of California, Los Angeles (UCLA), Los Angeles, CA, USA; ^2^Division of Hospitalist Medicine, UCSD, Rady Children's Hospital, San Diego, CA, USA; ^3^Rady Children's Hospital, San Diego, CA, USA; ^4^Division of Pediatric Infectious Disease, UCLA, Los Angeles, CA, USA; ^5^Department of Pediatrics, UCSD, Rady Children's Hospital, San Diego, CA, USA; ^6^UCSD Clinical and Translational Research Institute, San Diego, CA, USA; ^7^Pediatrics, UCSD School of Medicine, San Diego, CA, USA

## Abstract

*Background*. There is a need for increased palliative care training during pediatric residency.* Objective*. In this pilot study, we created a comprehensive experiential model to teach palliative care skills to pediatric residents. Our Comfort Care Modules (CCMs) address pediatric palliative care (PPC) topics of breaking bad news, dyspnea, anxiety, pain management, and the dying child. We also evaluated a scoring system and gathered qualitative data.* Methods*. The CCMs are part of the University of California San Diego pediatric residency's second-year curriculum. Comparisons were made for statistical trends between residents exposed to the modules (*n* = 15) and those not exposed (*n* = 4).* Results*. Nineteen of 36 residents (52%) completed surveys to self-rate their preparedness, knowledge, and confidence about PPC before and after the intervention. Resident scores increased in all areas. All improvements reached statistical significance except confidence when breaking bad news. Overall, the resident feedback about the CCMs was positive.* Conclusions*. This study demonstrates that the CCMs can be performed effectively in an academic setting and can benefit residents' self-perception of preparedness, confidence, and knowledge about pediatric palliative care. In the future, we plan to implement the modules on a larger scale. We encourage their use in interprofessional settings and across institutions.

## 1. Introduction

The importance of pediatric palliative and end-of-life care education programs for residents is increasingly evident [1, 2]. In the United States, more than 40,000 pediatric deaths occur each year from acute and chronic etiologies [3]. Palliative care is included as an optional subspecialty rotation to obtain educational units toward pediatric residency completion [4], but there is a concern that pediatric residents encounter these situations too infrequently to ensure skilled implementation of their pediatric palliative care (PPC) training [1, 5–9].

In response to the increased need for palliative care integration into the medical treatment plan, the American Academy of Pediatrics issued a comprehensive statement on palliative care for children living with a life-threatening or terminal illness [10]. Then, following a 2003 Institute of Medicine publication* When Children Die: Improving Palliative and End-of-Life Care for Children and Their Families*, an official hospice and palliative medicine fellowship training program was introduced [11]. The American Academy of Pediatrics Policy Statement regarding PPC advocates that general and subspecialty pediatricians, family physicians, pain specialists, and pediatric surgeons should be able to provide basic pediatric palliative and hospice care [2]. Web-based comprehensive training modules have been developed by the Initiative for Pediatric Palliative Care (IPPC) and Education in Palliative and End-of-Life Care (EPEC®) [12, 13]. Introduction of palliation earlier into patient care plans has been shown to increase child and family reported quality of life [14] as well as increase mean survival in adult patients [15]. Despite this heightened recognition, physicians and physicians-in-training perceive that residency programs in the United States have not adequately met their palliative care training expectations [5].

In order to increase our program's residents' exposure to PPC, we created comprehensive palliative care experiential training modules. It has been demonstrated that interactive educational training improves residents' performance and comfort with communication skills and breaking bad news [16, 17]; and procedural training exercises in cardiopulmonary resuscitation (CPR) improve residents' CPR skills [18–20]. We therefore created urgent, interactive mock-scenarios in palliative care that address the topics of breaking bad news (BBN), dyspnea and anxiety (DA), pain management (PM), and the dying child (DC). They were designed to reflect a level of expertise appropriate for a general pediatrician. In this study, our primary goal was to measure the effect of Comfort Care Modules (CCMs) on residents' self-perceived preparedness, confidence, and knowledge regarding pediatric palliative and end-of-life care.

## 2. Methods

### 2.1. Study Design

This is a single-center UCSD IRB-approved study involving pediatric and medicine-pediatric residents. We created Comfort Care Modules (CCMs) to simulate real-time urgent PPC scenarios. They are based upon the model of Pediatric Advanced Life Support (PALS) modules [21] but focus on PPC. The self-assessment measurements were modeled after previous measurements of PPC skills that are available in the literature [1, 22].

The CCM educational interactions transpire as follows: the scenario begins when investigators page one resident to a hospital room for a “mock comfort code.” The resident is met in the hallway in front of the room by a narrator (author Doshi) and informed that they are called to participate in a “mock comfort code.” The narrator hands the resident a written scenario, and the narrator has a copy to read aloud. The resident is instructed to consider this a “real-life” situation in the hospital and assume they have the usual staff, equipment, medication, and resources available to them. When the residents enter the hospital room, they encounter the actors who play the patient (author Ross) and/or parents (authors M. Pian and P. Pian) using a standardized script developed for each scenario. The BBN scenario requires only parents, the PM and DA scenarios require parents as well as the child, and the DC scenario utilizes parents and a mannequin patient. The resident communicates directly to the actors portraying the patient and/or parents. As in PALS scenarios, they are expected to take a history, obtain vital signs, perform a physical exam, and decide on the indicated actions and interventions. In regard to vital signs and physician exam results, the narrator provides the information if the resident inquires about it. The residents are allowed to use their palliative care handbook as a reference during the scenario, similar to PALS scenarios that utilize a resuscitation reference card. Residents receive the handbook at the start of their intern year and typically carry it on their person. If they do not have the handbook on the day of the CCM, we provide them with one. No actual patients, family members, examinations, or therapeutic actions are involved. Each CCM session continues until the resident reaches the predetermined goals of the scenario or the narrator felt that progress toward the goal terminated.

The residents are scored using scenario-specific checklists by two investigators per scenario to allow for interrater reliability. The score sheets are similar to the scoring instrument analyzed by Donoghue et al. for reliability and validity [23]. One point is assigned if the participant does not address the item, two points are assigned if the participant addresses the item after prompting from the actors and/or narrator, and three points are assigned if the participant addresses the issue without prompting. The actors use the score sheet checklist to guide standardized verbal feedback to the residents immediately following the module. No individual residents repeated any scenario in this pilot study.

Each CCM addresses competency in pharmacological and nonpharmacological interventions, communication, interdisciplinary teamwork, and assessment of goals of care. The modules of BBN, DA, PM, and DC were chosen by the investigators to be important core concepts in PPC as determined by experience and the literature [11, 24–26]. Complete scenario packets that include the resident scenario, full scenario (which serves as a script for the actors), and score sheets can be found in Supplement 1a–d (see Supplementary Material available online at https://doi.org/10.1155/2017/7568091). A brief summary of the scenarios and the goals are as follows.

#### 2.1.1. Breaking Bad News

The setting is the emergency department (ED). Part I: an otherwise healthy 10-month-old was found unresponsive while in the care of a babysitter and is receiving cardiopulmonary resuscitation. The resident is asked to speak to the family (who just arrived) about the situation and prognosis. Part II: the resuscitation effort was unsuccessful. The resident must inform the parents of their child's death.

*Goals*. Display empathetic and direct communication, avoid jargon and euphemisms, utilize a systematic approach to breaking bad news, allow for silence and time for parental response, and access available resources to provide the family with appropriate psychosocial and spiritual support.

#### 2.1.2. Dyspnea and Anxiety

The setting is the ED. The resident is called to assess a 16-year-old male with Duchenne muscular dystrophy. He receives home hospice care and was brought to the ED for respiratory distress and fever. The patient and family do not desire hospital admission.

*Goals*. Display empathetic and direct communication, recognize signs and symptoms of dyspnea and anxiety, provide appropriate pharmacologic and nonpharmacologic treatment, perform appropriate ongoing assessment for symptoms and response to intervention, and incorporate family-centered care and interdisciplinary collaboration into the care plan.

#### 2.1.3. Pain Management

The setting is the hematology-oncology unit. The admitting resident is called to assess a 10-year-old patient with relapsed acute lymphoblastic leukemia (ALL) status after two unsuccessful hematopoietic stem transplantations. The patient receives home hospice care and was admitted directly from home for intolerable pain. The patient and family do not desire hospital admission.

*Goals*. Display empathetic and direct communication, recognize signs and symptoms of nociceptive and neuropathic pain, provide appropriate pharmacologic and nonpharmacologic treatment, perform appropriate ongoing assessment for pain and response to treatment, and incorporate family-centered care and interprofessional collaboration into the care plan.

#### 2.1.4. The Dying Child

The setting is the intermediate care unit. The on-call resident is asked to assess a 12-year-old patient recently admitted with pneumonia. The patient has severe neurologic impairment due to anoxic brain injury at birth and cerebral palsy (Gross Motor Function Classification System Level 5), is fed exclusively by gastrostomy tube, and has a history of frequent, recurrent pneumonia. Despite broad-spectrum antibiotics and supplemental oxygen the patient is now moribund. The patient's code status is Allow Natural Death (AND).

*Goals*. Display empathetic and direct communication, recognize signs and symptoms of imminent death, provide appropriate pharmacologic and nonpharmacologic treatment for discomfort, minimize patient and family distress, and incorporate family-centered care and interdisciplinary collaboration into care plan.

The study period was November 8, 2011, to December 20, 2013. The CCMs were included as part of the resident education training curriculum for second-year pediatric and medicine-pediatric residents (*n* = 36). Because the CCMs were a component of the resident education curriculum and this was intended as a pilot study, we did not withhold the modules from individuals; therefore a sample of convenience rather than a randomized control design was utilized. Residents were informed by their program director at the beginning of the academic year that they are expected to participate in the CCMs unless they are engaged in direct patient care from which they are unable to excuse themselves in a safe manner. Comfort Care Modules were performed every 1–3 months with 1–3 modules per afternoon for available residents during their inpatient hospitalist, hematology-oncology, or pediatric intensive care unit (PICU) rotations. We rotated the comfort codes in order of BBN, PM, DA, and DC, depending on the availability of the investigators. No residents repeated a module (e.g., if the resident had already participated in a BBN, they would be exposed to a PM module).

Our primary outcome measures were based upon a 29-question electronic survey administered via SurveyMonkey® (https://www.surveymonkey.com) before and after residents' second year of training. The survey was designed to take less than five minutes to complete and was modeled after PPC surveys for physician trainees from the literature [1, 22]. The scores were based on Likert scales of 1–5 for feeling of preparedness (1 = strongly disagree and 5 = strongly agree) and scales of 1–5 for feeling of confidence and knowledge (1 = poor and 5 = excellent). We examined whether there was improvement in an individual's postintervention survey scores. We took into consideration their exposure to palliative care lectures or the number of days between module exposure and postsurvey administration. A question addressing a topic that was not addressed by our modules (organ donation) served as a surrogate control question. We also measured the residents' scores on each module as well as the scorers' interrater reliability.

For qualitative data collection, the intervention group participants completed an electronic survey after each module that included open-ended questions about the CCMs. The qualitative information gathered from this survey was categorized and quantified to determine what residents perceived as positive about the modules and what aspects of the modules could be improved.

### 2.2. Statistical Analysis

Data from participants who completed the pre- and postsurveys were analyzed. For comparison within the intervention group we utilized a paired *t*-test; and for comparison between groups we utilized a two-sample *t*-test for unequal variance. Comparison between the two groups is limited because of the small size of the nonintervention group. Spearman's correlation coefficient evaluated the relationship between postsurvey scores and the number of PPC noon conferences attended and days between intervention and postsurvey administration. For the intervention group, we documented their CCM scores. The interrater reliability of the scorers of the scenario score sheet was determined by interclass correlation (ICC). We used leaner mixed effects models to evaluate if the number of PPC noon lectures attended or the number of CCMs affected the module score. We used fixed effects and random intercept to account for within subject correlation.

## 3. Results

### 3.1. Descriptive Statistics

Twenty-eight pediatric and eight medicine-pediatric residents received an electronic survey invitation at the beginning and end of the academic year. Twenty-four pediatric and seven medicine-pediatric residents returned either a pre- or postsurvey. Sixteen pediatric and three medicine-pediatric residents (53%) returned both a pre- and a postsurvey. Of the 19 total respondents, 15 were exposed to the CCMs during their rotations (intervention group) and four were not (nonintervention group). The descriptive data obtained from the intervention and nonintervention groups were similar, except that more residents in the nonintervention group had PPC exposure prior to the study period (Table 1). In the intervention group, there was no relationship between composite scores and the number of days neither between the postsurvey administration and the intervention nor with the number of modules completed. There was a small trend toward an increase in the pre/postsurvey score difference for those with more PPC noon conference attendance prior to the study period (*ρ* = 0.382, *p* = 0.160).

### 3.2. Module Scoring and Performance

The average length of CCMs, including debriefing, was 21 ± 6 minutes. The BBN scenario was completed in 27 ± 3 minutes, DA in 15 ± 5 minutes, PM in 19 ± 7 minutes, and DC in 25 ± 1 minutes.

The average score for the BBN scenario was 69.2%  ±  8.0%, for DA 79.4%  ±  6.5%, for PM 76.7%  ±  4.6%, and for DC 71.5%  ±  8.5%. There were a total of eight raters over the course of the study, with two raters assigned to each module. The overall concordance (interclass correlation) between all modules was 0.88 (95% CI: 0.48–0.90). We did not find any statistically significant effect on modules scores for the number of modules performed (*β* = 0.019, *p* = 0.264) or the number of noon conferences attended (*β* = −0.027, *p* = 0.378).

### 3.3. Self-Perception of Preparedness, Knowledge, and Confidence

#### 3.3.1. Quantitative Data

**Figure 1:**
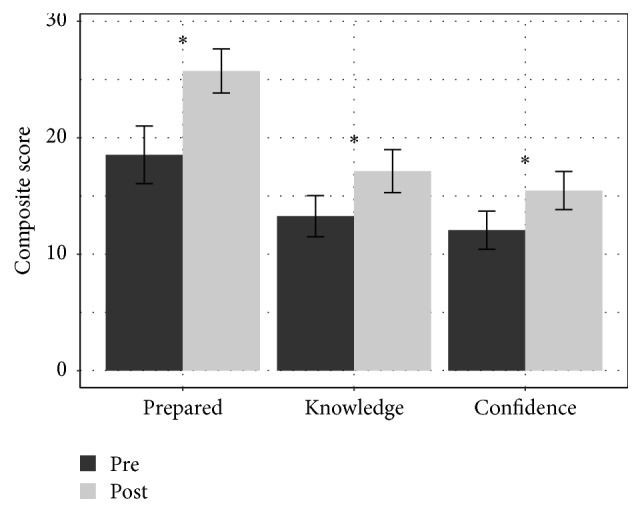
Intervention group (*n* = 15) pre- and postcomposite survey scores measuring feelings of preparedness, knowledge, and confidence regarding questions related to general pediatric palliative and end-of-life care. *∗* indicates significance of *p* > 0.05.

**Figure 2:**
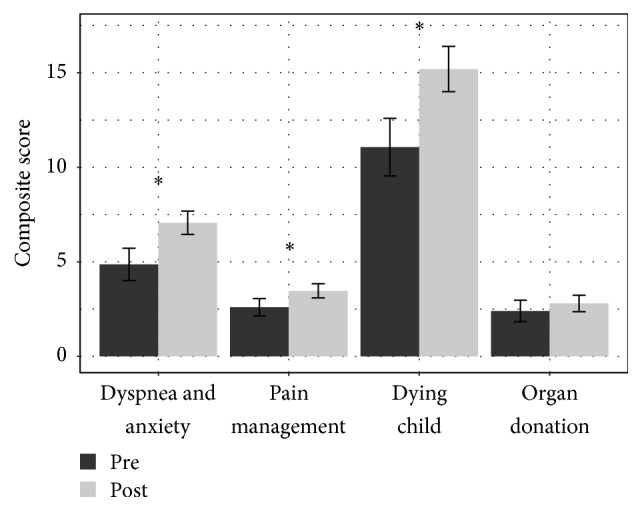
Intervention group (*n* = 15) pre- and postcomposite survey scores of feeling prepared to address pediatric palliative and end-of-life care by Comfort Care Module topic (dyspnea and anxiety, pain management, and dying child). A question regarding organ donation (not specifically addressed by our scenarios) was included for comparison as a surrogate control. *∗* indicates significance of *p* > 0.05.

Following exposure to the modules, the intervention group had statistically significant overall survey score increases in all categories (Figure 1). The nonintervention group survey scores showed a small, statistically insignificant, trend to increased scores. Survey questions not directly addressed by the modules such as organ donation showed less increase than that observed for topics specifically addressed by the CCMs (Figure 2). When individual questions were analyzed relating to specific topics addressed by the CCM modules, all dimensions showed significant increase in composite postscores except for confidence breaking bad news (Table 2). There were no significant correlations between CCM score and postmodule survey (raw or delta) score.

#### 3.3.2. Qualitative Data

**Figure 3:**
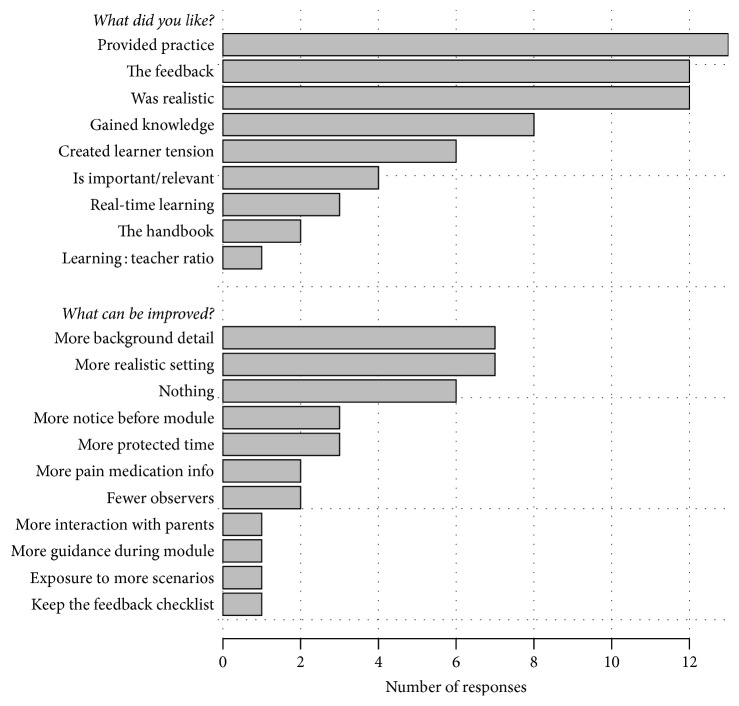
Summary of free-text responses to open-ended questions about the scenarios of “what did you like about the module?” and “what can be improved about the module?” following completion of a Comfort Care Module.

Overall, the residents felt the CCMs provided useful practice and were realistic, and they appreciated the feedback portion of the modules. Their remarks were positive toward the handbook; we did not quantify how often they reference the handbook during their actual practice of patient care. Areas of module improvement identified were that residents wanted more detail about what resources they had available in the scenario, more realistic equipment such mannequins with real-time vitals, and more advanced notice to protect their time to attend the modules (Figure 3).

## 4. Discussion

Residency programs use methods such as lectures and/or lecture-based role-play to teach palliative and end-of-life care; however, these methods often may not meet trainees' needs [1]. Hands-on skill modules and procedural training exercises, such as the PALS program, improve residents' skills and performance significantly in the short-term with mixed results on long-term skill recall [18, 27]. Positive results were achieved when trainees were taught how to break bad news utilizing standardized patients [28, 29] but, to our knowledge, experiential programs such as our CCMs that address a broad range of PPC topics have not been studied in detail.

Our results suggest that procedural training exercises such as CCMs can improve residents' self-rated preparedness, knowledge, and confidence in PPC and are accepted by the residents as a learning tool. Barriers to implementation of mock “comfort code” scenarios are time constraints due to the demanding schedule of the residents and the team implementing the modules as well as costs to provide standardized patients and interactive equipment. Recognizing the potential expense of modules performed with professional standardized patients, we designed the CCMs to be detailed and readily adaptable for educators to role-play without relying on standardized patients. We did not want the lack of standardized patients to preclude programs from implementing this format of palliative care education.

The nonintervention group was small; however, we included the group to illustrate statistical trends. The residents' survey scores improved more in the group that participated in the CCMs than in the group that did not. Scores improved in areas specifically addressed by the CCMs more so than topics not specifically addressed by the modules such as organ donation. Among all residents, there was a weak positive correlation between the number of PPC lectures attended and an improvement in survey score, suggesting that lecture-based palliative care education may provide additional benefit. The CCM group's self-confidence regarding PPC improved the least of all dimensions. One explanation for this is that participation in a CCM makes residents more aware of their knowledge gaps. In addition, residents may judge themselves more critically than do others.

This pilot study indicated to us that the positive trend in postsurvey scores encourages further study of the interactive CCM program. Areas to address in the future will be to eliminate sources of bias. Recall bias is present when residents report past exposure to PPC, so this could be proactively tracked in the future. Bias exists when the authors, whom the residents are supervised by, are the actors. It is also possible that residents may fill out the post-PPC survey in a positive fashion to please the authors or adjusted their responses if there was concern that the actors (authors) would judge them on their responses. There also may be unintentional bias on the part of the scorer if they had previously interacted with the resident; two scorers were used to address this possibility. In the future, utilizing standardized patients could provide residents with more realistic scenarios, allow for objective measurement of their PPC skills rather than self-perceived assessment, and reduce bias. Other opportunities for objective measurement will be resident participation in each module twice and the administration of concrete knowledge-based testing as an outcome measure.

## 5. Conclusion

Our study suggests that the Comfort Care Module program is a useful approach toward improving pediatric resident self-rated preparedness and knowledge about pediatric palliative and end-of-life care. Based on their narrative feedback, pediatric residents report many positive aspects of the CCM experience. Future directions include a larger randomized trial within and across institutions utilizing standardized patients, training multidisciplinary team members (nurses, nurse practitioners, physician assistants, etc.), and execution in different departments (emergency department, hematology-oncology, intensive care unit, etc.). We chose to openly share these standardized modules with the academic community with the goal of fostering palliative care education for residents and to motivate similar educational efforts for interprofessional teams.

## Supplementary Material

The Supplementary Materials contain the complete scenario packets for the Breaking Bad News, Dyspnea and Anxiety, Pain Management, and the Dying Child Comfort Care Modules. This includes the resident scenarios, full scenarios (which serves as a script for the actors), and score sheets.

## Figures and Tables

**Table 1:** Descriptive data: intervention versus nonintervention groups.

Variable	*N*	Percent total
Intervention group	15	
Male	6	40%
Female	9	60%
Pediatrics	14	93%
Medicine-Pediatrics	1	7%
Exposure to pediatric palliative care teaching (>2 noon conferences in the past year)	5	33%
Exposure to pediatric palliative care discussions (>2 in the past year)	7	47%
Initiated pediatric palliative care discussion (>2 in the past year)	1	20%

Variable	*N*	Percent total

Nonintervention group	4	
Male	2	50%
Female	2	50 %
Pediatrics	2	50%
Medicine-Pediatrics	2	50 %
Exposure to pediatric palliative care teaching (>2 noon conferences in the past year)	2	50%
Exposure to pediatric palliative care discussions (>2 in the past year)	3	75%
Initiated pediatric palliative care discussion (>2 in the past year)	3	75%

**Table 2:** Intervention group (*n* = 15) pre- and postsurvey averaged scores of feeling knowledgeable and confident about pediatric palliative and end-of-life care by topic. Individual questions were answered using a Likert scale (1 = poor and 5 = excellent). Scores are presented as means (standard deviations). Statistical significance is determined by a paired *t*-test. ^*∗*^Significant difference <0.05.

	Prescore	Postscore	Delta	*p* value
Knowledge				
Breaking bad news	2.37 (0.85)	2.93 (0.75)	0.57 (0.70)	0.008^*∗*^
Dyspnea and anxiety	1.67 (0.62)	2.73 (0.70)	1.07 (0.96)	<0.001^*∗*^
Pain management	1.87 (0.64)	2.87 (0.64)	1.00 (0.76)	<0.001^*∗*^
Dying child	2.50 (0.76)	2.83 (0.67)	0.33 (0.49)	0.019^*∗*^
Confidence				
Breaking bad news	2.13 (0.67)	2.70 (0.62)	0.57 (0.59)	0.002^*∗*^
Dyspnea and anxiety	1.60 (0.63)	2.47 (0.74)	0.87 (0.83)	0.001^*∗*^
Pain management	1.87 (0.64)	2.60 (0.74)	0.73 (0.70)	0.001^*∗*^
Dying child	2.17 (0.72)	2.50 (0.57)	0.33 (0.62)	0.055
