# Supplementary material for: Interactive Palliative and End-of-Life Care Modules for Pediatric Residents
Source: Int J Pediatr. 2017 Feb 12;2017:7568091. doi: 10.1155/2017/7568091 (PMC5329665; doi:10.1155/2017/7568091)
Supplement: Supplementary file 1 — The Supplementary Materials contain the complete scenario packets for the Breaking Bad News, Dyspnea and Anxiety, Pain Management, and the Dying Child Comfort Care Modules. This includes the resident scenarios, full scenarios (which serves as a script for the actors), and score sheets. [file 7568091.f1.pdf]

Scenario #1

This is a Comfort Code Module. We ask that you treat the scenario as real and immerse yourself in the role. You have all resources available in a hospital setting.

Setting

Emergency Department (ED) Hallway.

Scenario

A 10-month-old previously healthy female who was being cared for by a babysitter at home was brought in by paramedics with cardiopulmonary resuscitation (CPR) in progress after being found unconscious and not breathing for an unknown period of time. The fellow and attending have been in the trauma bay attempting cardiopulmonary resuscitation for 60 minutes and she remains pulseless. The attending physician asks that you update the parents that because resuscitation has been unsuccessful for over 1 hour, she may die. If she survives, she will most likely be severely neurologically impaired.

The parent(s) were called in by an ED staff member and have been told *only* that their child was brought to the ED via ambulance. You now go to meet the parent(s) in the hallway to update them.

Once you have delivered the news, you will excuse yourself to check on the patient's status when appropriate (narrator will update you) and you will return to update the parent(s).

Scenario #1: Breaking Bad News

Scenario Goals

1) Display empathetic and direct communication. Avoid medical jargon and euphemisms.
2) Utilize a systemic approach to breaking bad news.
3) Allow silence and time for parental response.
4) Access available resources to provide family with psychosocial and spiritual support.
5) Provide clear information about patient's current medical status and prognosis.

Roles

Parent(s)
Narrator
Scorer(s)

Setting

Emergency Department (ED) Hallway

Narrator (read aloud all text NOT in parentheses)

This is a Comfort Code Module. We ask that you treat the scenario as real and immerse yourself in the role. You have all resources available in a hospital setting.

(Part I): A 10-month-old previously healthy female who was being cared for by a babysitter was brought in by paramedics with cardiopulmonary resuscitation (CPR) in progress after being found unconscious and not breathing for an unknown period of time. The fellow and attending have been in the trauma bay attempting cardiopulmonary resuscitation for 60 minutes and she remains pulseless. The attending physician asks that you update the parents that because resuscitation has been unsuccessful for over an hour, she may die. If she survives, she will most likely be severely neurologically impaired.

The parent(s) were called in by an ED staff member and have been told *only* that their child was brought to the ED via ambulance. You now go to meet the parent(s) in the hallway to update them. Once you have delivered the news, you will excuse yourself to check on the patient's status when appropriate (I will update you) and you will return to update the parent(s)

(Part II: After parent(s) ask learner to check on patient) The attending physician and fellow stopped the resuscitation. They ask you to let the parent(s) know that the patient has died.

Part I

Once parent(s) see learner, demand: "What happened?" "What's going on?"

Learner	Parent(s) Response
**If**	
Does not offer to move locations	"Isn't there somewhere more quiet to go?"
Delivers the bad news	Parent(s) potential reactions: Sobbing, silent/stoic, angry, agitated, etc.
Does not offer update	"Please find out what's going on now."
Offers to bring family to watch the code	"No, we can't bear to see."

Part II

Parent(s) look somewhat hopeful, but overall still sad and upset

Learner	Parent(s) Response
**If**	
Does not offer to bring family to patient after she died	"What do we do now?" "Can we see her?"
Does not offer psychosocial support	"Is there someone we can talk to?" "Is there a chaplain?" "Can we call our family members?"

Endpoint

The scenario is complete when the learner communicates that patient died, offers to take parent(s) to the child and offers psychosocial support.

Scenario #1: Breaking Bad News

Date: _________ Completed Pre-Palliative Care Survey (Yes/No) _____________

Learner's Name: ____ Scorer's Name: ____________________________________

Role Player's Names/Roles: ___________________________________ Narrator's Name: ___________________________________

Time Paged: _______ Time Arrived: ________ Time Debriefing Completed: _________

Critical Performance Items	Examples	SCORING<br>**Please circle a score.**<br>1=Did not complete<br>2=Completed with prompting<br>3=Completed with NO prompting
**General**		
Identifies self and role in patient's care		1.................2.................3
Empathetic and direct communication with parent(s)	Places parent(s) at ease<br>Uses non-judgmental statements<br>Listens to parent(s) effectively<br>Acts professionally	1.................2.................3
**Part I**		
Creates an appropriate setting	Offers to move to a quiet space, offers to sit, turns off pager, etc.	1.................2.................3
Finds out what parent knows	Asks what parent(s) have been told	1.................2.................3
Fires a "warning shot"	Informs parent(s) that what they are going to say next will be unpleasant/bad news leading in with phrases such as:<br>"I wish I had better news but…"<br>"I'm afraid I have bad news…" etc.	1.................2.................3
Responds appropriately/empathetically to parental emotions<br>*Score "1" if implies to know how parent(s) feel(s)*	Facial expressions, words, body language, Does not say "I understand"<br>Acknowledges, validates, listens	1.................2.................3
Is silent, allows time for parental response	Permit silence, pause between sentences/thoughts, allows emotion	1.................2.................3
Avoids jargon and euphamisms	No large/unfamiliar words or medical terms<br>No euphamisms	1.................2.................3
**Part II**		
Creates an appropriate setting	Offers to move to a quiet space, offers to sit, turns off pager, etc.	1.................2.................3
Fires a "warning shot"	Informs parent(s) that what they are going to say next will be unpleasant/bad news leading in with phrases such as:<br>"I wish I had better news but…"<br>"I'm afraid I have bad news…" etc.	1.................2.................3
Responds appropriately/empathetically to parental emotions<br>*Score "1" if implies to know how parent(s) feel(s)*	Facial expressions, words, body language, Does not say "I understand"<br>Acknowledges, validates, listens	1.................2.................3
Is silent, allows time for parental response	Permit silence, pause between sentences/thoughts, allows emotion	1.................2.................3
Avoids jargon and euphemisms<br>*Score "1" if does not say form of word "die"*	No large/unfamilliar words or medical terms<br>States form of the word "die"	1.................2.................3
**Case Conclusion**		
Offers to take parent(s) to see patient		1.................2.................3
Offers psychosocial support	Suggests at least one: social worker, child life, chaplain, religious/spiritual representative from community, family members, friends. Full credit even if deferred to social worker.	1.................2.................3

Scenario #1: Breaking Bad News p.2

	Check ONE Only				
	Unsatisfactory	Below Average	Average	Above Average	Superior
	1	2	3	4	5
Confident					
Comfortable					
Empathetic/Sensitive					
Respectful/Professional					
Informative					
Comforting					

Comments:

Scenario #2

This is a Comfort Code Module. We ask that you treat the scenario as real and immerse yourself in the role. You have all resources available in a hospital setting.

Setting

Emergency Department (ED), bedside

Scenario

A 16-year-old male with Duchenne muscular dystrophy is brought to the ED by his parent(s) because he is experiencing shortness of breath. He is receiving hospice care at home. Today he started to breathe faster than usual. The patient, who can speak, is not in pain but is very uncomfortable and states that it feels difficult to breathe. You go to see the patient.

Scenario #2: Dyspnea and Anxiety

Scenario Goals

1) Display empathetic and direct communication.
2) Recognize signs and symptoms of dyspnea and anxiety.
3) Provide appropriate pharmacologic and non-pharmacologic treatment.
4) Perform appropriate ongoing assessment of symptoms and response to treatment.
5) Incorporate family-centered care and interdisciplinary collaboration into care plan.

Roles

Patient
Parent(s)
Narrator
Scorer(s)

Setting

Emergency Department (ED), bedside

Narrator (read aloud)

This is a Comfort Code Module. We ask that you treat the scenario as real and immerse yourself in the role. You have all resources available in a hospital setting.
A 16-year-old male with Duchenne muscular dystrophy is brought to the ED by his parent(s) because he is experiencing shortness of breath. He is receiving hospice care at home. Today he started to breathe faster than usual. The patient, who can speak, is not in pain but is very uncomfortable and states that it feels difficult to breathe. You go to see the patient.

Background (for narrator reference only, not read aloud)

A 16-year-old male with Duchenne muscular dystrophy is brought to the ED by his parent(s) because he is experiencing shortness of breath. He uses nocturnal BiPAP at home, but frequently asks that it be removed because he dislikes it. He receives hospice care at home and the family has discussed goals of care many times with his medical team. The patient and his parent(s) have stated that they understand the terminal nature of the patient's illness and wish for Allow Natural Death (AND) status and that he die at home. They brought in a Physician Orderd for Life-Sustaining Treatment (POLST) form. At home, the parent(s) was(were) unsure how to proceed when he developed respiratory distress and appeared uncomfortable.
The patient, who can speak, is not in pain but is very uncomfortable and states that it feels difficult to breathe. At baseline, his anxiety level is a “2” out of “10”.

Notes for Scenario:

Code Status

Allow Natural Death (AND): No intubation. No chest compressions. No cardiac medications. POLST form available.
Limited additional medical interventions: Yes (antibiotics and supplemental oxygen).

Home medication

Morphine 8mg PO q 2-4 hours as needed (no recent use)

IV Access

None

Physical Exam

Learner	Narrator Response
**If**	
Asks for vitals	Wt: 55 kg T 39 HR 125 RR 40 BP 126/85 O2 88% on RA<br>Pain Score: 0<br>Anxiety: 5 out of 10
Asks for physical exam	Gen: Anxious appearing<br>Resp: Nasal flaring, shallow breaths, crackles in R middle lobe. Decreased breath sounds bilaterally. Prominent abdominal movement with respirations<br>CV: Tachycardic, no murmurs, rubs or gallop<br>Ext: Warm, well-perfused, cap refill < 2 seconds

Scripted Responses

Patient

Short of breath, worried, and unable to move without assistance. “I’m scared,” “What’s happening?” “I can’t breathe!”

Learner	Patient Response
**If**	
Suggests lab draw or BiPAP	“No!”
Suggest hospital admission	“I want to go home!” “I hate the hospital!” “Am I dying?”
Gives Morphine (no Ativan)<br>Gives Ativan (no Morphine)<br>Gives Morphine and Ativan	“I can breathe a little better, but still don’t feel great”<br>“I feel a little better, but still feel like I can’t breathe”<br>“I am feeling better”

Parent(s)

Scared and unsure of what is happening with their son. States for the provider to “do something!” Parent(s) realizes the disease process is terminal and are afraid he might die today. Parent(s) does not want him to suffer. Although parent(s) brought him into the ED the parent(s) can be refocused on his palliative goals if addressed appropriately.

Learner	Parent Response
**If**	
Suggests IV/labs/VBG	"Why do you want to do that?"<br>“We do not want any invasive procedures, we want him to be comfortable.”
Suggests medication for anxiety/dyspnea	“Is he going to become addicted?”<br>“Is he going to stop breathing?”
Suggests chest X-ray	They do not refuse but ask, “Do you really need one?”
Suggests hospital admission	"No, I(we) can care for him at home. He is more comfortable there

Response to interventions:

- Supplemental O2 will increase the patient’s O2 saturation to 92%, but will not relieve his distress.
- An antipyretic will help patient become more comfortable (T 37, RR 30, HR 100, BP 110/85)
- An opiate and anxiolytic is required to make the patient more comfortable.
  - Opiate alone will improve the respiratory distress, but not completely alleviate it (RR 30, HR 100, BP 110/85).
  - Anxiolytic alone will help patient be less anxious, but will remain short of breath. (RR 30, HR 100, BP 110/85).
  - If opiate and anxiolytic provided, the vitals are: RR 24, HR 90, BP 110/85 and appears calm.
- Adjuvant Methods: He states he is more comfortable. He especially responds to fan blowing in the face.

Endpoint

The scenario ends when the patient is comfortable and a conversation regarding appropriate disposition of the patient is started.

Scenario #2: Dyspnea and Anxiety

Date: _________ Completed Pre-Palliative Care Survey (Yes/No) _____________

Learner's Name: ____ Scorer's Name: ____________________________________

Role Player's Names/Roles: ____________________________________ Narrator's Name: __________________________________

Time Paged: _______ Time Arrived: ________ Time Debriefing Completed: _________

Critical Performance Items	Examples	SCORING<br>**Please circle a score.**<br>1=Did not complete<br>2=Completed with prompting<br>3=Completed with NO prompting
**General**		
Identifies self and role in patient's care		1.................2.................3
Empathetic and direct communication with parent(s)	Places parent at ease<br>Uses non-judgmental statements<br>Listens to parent effectively<br>Acts professionally	1.................2.................3
Empathetic and direct communication with patient	Places patient at ease<br>Uses non-judgmental statements<br>Listens to patient effectively<br>Acts professionally	1.................2.................3
**Patient Management**		
Obtains vitals, performs physical exam	*MUST do both for any credit*	1.................2.................3
Addresses code status/POLST form		1.................2.................3
Addresses patient and parental concerns about goals of care.	Clarifies parent's/patient's desire for invasive treatment (IV, labs, intubation, antibiotics).<br>Acknowledge family's distress and fears	1.................2.................3
Provides 2 or more pharmacologic treatments (MUST *offer oxygen for any credit*)	Morphine<br>≥ 0.1mg/kg PO (*credit*: 5-15mg)<br>≥ 0.05 to 0.1 mg/kg SQ (*credit*: 2.5-10mg)<br>Lorazepam<br>0.05mg/kg/dose PO/SL/IV<br>Diazepam<br>0.03 mg/kg PO, one dose<br>Supplemental oxygen via nasal cannula	1.................2.................3
Provides 2 or more non-pharmacologic treatments	Fan face<br>Repositioning<br>Calm environment, reduce noise<br>Massage therapy<br>Facilitates family expression of fears and emotions<br>Exploration of spiritual needs<br>Calls for staff who knows family well	1.................2.................3
**Case Conclusion**		
Discusses home plan and follow-up *(Suggest at least 1 for any credit)*	Offers social work, hospice, spiritual/ religious support, caregiver support resources, and other multidisciplinary avenues	1.................2.................3

Scenario #2: Dyspnea and Anxiety p.2

	Check ONE Only				
	Unsatisfactory	Below Average	Average	Above Average	Superior
	1	2	3	4	5
Confident					
Comfortable					
Empathetic/Sensitive					
Respectful/Professional					
Informative					
Comforting					

Comments:

Scenario #3

This is a Comfort Code Module. We ask that you treat the scenario as real and immerse yourself in the role. You have all resources available in a hospital setting.

Setting

Hematology-Oncology Unit, bedside

Scenario

A 10-year-old female with relapsed acute lymphoblastic leukemia (ALL), status-post second bone marrow transplant (BMT), is on home hospice and was directly admitted from home with worsening leg pain, back pain and nausea. Patient is alert, developmentally appropriate and active in her own care. Patient rates the pain as a 20 out of 10. Parent(s) is(are) upset to see the child in so much pain. You go to see the patient.

Scenario #3: Pain Management

Scenario Goals

1) Display empathetic and direct communication.
2) Recognize signs and symptoms of nociceptive and neuropathic pain.
3) Provide appropriate pharmacologic and non-pharmacologic treatment.
4) Perform appropriate ongoing assessment of symptoms and response to treatment.
5) Incorporate family-centered care and interdisciplinary collaboration into care plan.

Roles

Patient
Parent(s)
Narrator
Scorer(s)

Setting

Hematology-Oncology Unit, bedside

Narrator (read aloud)

This is a Comfort Code Module. We ask that you treat the scenario as real and immerse yourself in the role. You have all resources available in a hospital setting.
A 10-year-old female with relapsed acute lymphoblastic leukemia (ALL), status-post second bone marrow transplant (BMT), is on home hospice and was directly admitted from home with worsening leg pain, back pain and nausea. Patient is alert, developmentally appropriate and active in her own care. Patient rates the pain as a 20 out of 10. Parent(s) is(are) upset to see the child in so much pain. You go to see the patient.

Background (for narrator reference only, not read aloud)

A 10-year-old female with relapsed acute lymphoblastic leukemia (ALL), status-post second bone marrow transplant (BMT), is on home hospice and was directly admitted from home with 2 weeks of worsening leg pain, back pain and nausea. Patient has been using 2 Vicodin every 4 hours for the past 5 days which has not alleviated the pain. The pain is described as intermittent, aching, burning, and sometimes radiating down both legs. Alleviating factors are heating pads and leg massage. Patient is refusing to walk and rates the pain a 20 out of 10. Patient is complaining of nausea from the Vicodin. Patient is alert, developmentally appropriate and active in her own care. There are no new stressors but she seems more sullen than usual. Parent(s) is(are) present and upset to see the child in so much pain.

NOTES FOR SCENARIO:

Pain Medications

Ibuprofen 400mg PO every 6 hours prn. Due to pain. Has taken maximal ordered dosing for the last 5 days.
Hydrocodone/Acetaminophen (Vicodin) 10mg/300mg PO every 4 hours prn pain not controlled by ibuprofen. Has taken maximal ordered dosing for the last 5 days.
Ondansetron (Zofran) 8mg PO every 8 hours prn nausea

IV Access

Broviac

Pain Description

Severity: 20 out of 10
Location: Both legs and back
Duration: 2 weeks, worsening
Quality: Achy and burning pain that sometimes radiates down both legs
Frequency: Constant ache. Burning pain 2-3 times per day lasting hours at a time
Alleviating Factors: Improved by heating pads and leg massage. Vicodin every 4hrs helped initially, but not currently
Exacerbating Factors: Movement (refusing to walk)

Tolerable Level of Pain for Patient

6 out of 10

Code Status

Full Code

Physical Exam

Learner	Narrator Response
**If**	
Asks for vitals<br>*See below "Response to Pain Medication"	Wt 25kg T 37 HR 130 RR 35 BP 115/85<br>O2 saturation 99% on room air<br>Pain Score: 20 out of 10
Asks for Physical Exam	Gen: Thin, furrowed brow/grimacing, whining, moaning, scared
	Resp: Clear breaths CV: Tachycardic, no murmurs, rubs or gallops<br>Ext: Warm well-perfused, cap refill < 2 seconds. Not moving legs.

Scripted Responses

Patient

Moans in pain and frequently says "It hurts." States: "I'm scared" and "What's happening to me?" She has become sad and more withdrawn over the past few months because she is scared of dying and tired of being sick.

Learner	Patient Response
**If**	
Suggests opiates	"Vicodin wasn't working!"<br>"Vicodin makes my stomach feel funny"

Parents

Concerned and upset for their child. Sad that she is facing another setback. Parent(s) want pain control for the child, but become(s) nervous and hesitant when opiates are mentioned. One parent recently lost his(her) mother to lung cancer-- soon after morphine was used regularly for her pain, she died. The parent(s) realize the child's disease process is terminal but is(are) having difficulty coming to terms with it. However, the patient has been through enough treatments, chemotherapy and BMTs and the parent(s) does(do) not wish for their child to suffer any more.

Learner	Parent Response
**If**	
Wants labs or testing to diagnose cause of pain	"Why do you want to do that?<br>"We know the cancer is getting worse."<br>"We just want her to be comfortable"
Offers chemotherapy or radiation	"Too many side effects." "She's been through enough."
Suggests opiates to control pain	"Is she going to become addicted?" "Is she going to die?" "Do we have to give that?" Takes learner aside to tell about patient's grandmother.
Insists on keeping patient in hospital	"No, we can care for her at home, she is more comfortable there"
Does NOT offer long-term plan once pain is controlled	"How will we control her pain at home?" "What about her nausea?"

*Response to pain medication

- Every time dose of IV opiate given at equianalgesic dose of at least 10mg PO Vicodin --> scene advances 30 minutes
- After first equianalgesic opiate dose, patient states pain is a 10. HR 120, RR 30. Learner should repeat dose –> scene advances 30 minutes
- After second equianalgesic opiate dose, patient states pain is an 8. HR 110, RR 25. Learner should repeat dose –> scene advances 30 minutes
- After third equianalgesic opiate dose, patient states pain is a 6. HR 100, RR 22. Learner should elicit that patient is comfort able with this level of pain

Endpoint

The scenario is complete when patient's pain is a 6 out of 10 and resident discusses long-term plan

Scenario #3: Pain Management

Date: ___________________________

Resident's Name: ____ Scorer's Name: ____

Role Player's Names: ____

Time Paged: _______ Time Arrived: ________ Time Debriefing Completed: _________

Critical Performance Steps	Descriptions	SCORING<br>**Please circle a score.**<br>1=Did not complete<br>2=Completed with prompting<br>3=Completed with NO prompting
**General**		
Identifies self and role in patient's care		1.................2.................3
Empathetic and effective communication with parent	Places caregiver at ease<br>Uses non-judgmental statements<br>Listens to caregiver effectively<br>Acts professionally	1.................2.................3
Empathetic and effective communication with patient	Places patient at ease<br>Uses non-judgmental statements<br>Listens to patient effectively<br>Acts professionally	1.................2.................3
**Patient Management**		
Addresses code status/POLST form		1.................2.................3
Obtains vitals, performs physical exam	*MUST do both for any credit*	1.................2.................3
MUST ask all 6 for any credit, otherwise score 1	Severity, Location, Duration,<br>Quality, Frequency, Alleviating/Exacerbating Factors	1.................2.................3
Assesses tolerable pain level for patient	6 or below is tolerable	1.................2.................3
Determines home medication regimen	<u>Hydrocodone</u> (Vicodin) 2 tabs (10mg)<br>q4hrs x last 5 days<br><u>Ibuprofen</u> 400mg every 8 hours<br><u>Ondansetron</u> 8mg every 8 hours prn nausea	1.................2.................3
Determines opiate start dose based on home meds<br>*MUST give opiate (at indicated dose) for any credit*<br>*MUST ask about home meds for any credit*	<u>Morphine</u> 3mg IV is equivalent dose OR<br><u>Hydromorphone</u> (Dilaudid) 0.6 mg IV<br>is equivalent dose	1.................2.................3
Asks about non-pharmacologic treatments<br>*MUST ask 1 for any credit*	Inquires what worked in past, offers massage, healing touch, acupuncture, heat, cool packs	1.................2.................3
Identifies neuropathic pain	Specifically identifies neuropathic pain or suggests medication such as Gabapentin or Amitriptyline	1.................2.................3
Addresses patient and parental concerns regarding opiates<br>*MUST ask 1 for any credit*	State that patient is not dying<br>State that opiates will not kill patient<br>State that the opiates are needed to treat patient's pain<br>Address concerns of respiratory depression	1.................2.................3
**Case Conclusion**		
Develops Home/Long-term plan<br>*MUST mention 2 (including scheduled opiate) for any credit*	Scheduled opiate<br>Patient-controlled analgesia (PCA)<br>Plan for breakthrough pain<br>*Adjuvant medications:*<br><u>Constipation</u>- Polyethylene glycol, senna, bisacodyl<br><u>Anxiety</u> - Diazepam<br><u>Depression</u> - Amitriptyline, SSRI, SNRI<br><u>Gastritis</u> - Ranitidine, omeprazole, calcium carbonate<br><u>Nausea</u> - ondansetron, metaclopramide, prochlorperazine	1.................2.................3

Scenario #3: Pain Management p.2

	Check ONE Only				
	Beginner	Novice	Developing	Near Competent	Competent
	1	2	3	4	5
Confident					
Comfortable					
Empathetic/Sensitive					
Respectful/Professional					
Informative					
Comforting					

Comments:

Scenario #4

This is a Comfort Code Module. We ask that you treat the scenario as real and immerse yourself in the role. You have all resources available in a hospital setting.

Setting

Intermediate Care (”step-down”) Unit, bedside

Scenario

A 12-year-old male (drowning at age 4 with severe neurologic impairment, cerebral palsy: gross motor function classification system level 5, severe scoliosis, exclusively fed via gastrostomy tube and history of recurrent pneumonias over the past year) was admitted from home hospice three days ago for multi-lobar pneumonia, respiratory distress and increasing secretions. The family brought his Physician Orders for Life-Sustaining Treatment (POLST) form indicating Allow Natural Death (AND). The most recent chest x-ray revealed a “white-out” of both lung fields and is not responding to broad-spectrum antibiotics.

You are called to assess the patient because the IV is out, he “doesn’t look good” and a dose of antibiotics is due. You go to see the patient.

Scenario #4: The Dying Child

Scenario Goals

1) Display empathetic and direct communication.
2) Recognize signs and symptoms of imminent death.
3) Provide appropriate pharmacologic and non-pharmacologic treatment for pain, discomfort and "death rattle."
4) Perform appropriate ongoing assessment of comfort measures and response to treatment.
5) Incorporate family-centered care and interdisciplinary collaboration into care plan.

Roles

Patient (may use mannequin)
Parent(s)
Narrator
Scorer(s)

Setting

Intermediate Care ("step-down") Unit, bedside

Narrator (read aloud)

This is a Comfort Code Module. We ask that you treat the scenario as real and immerse yourself in the role. You have all resources available in a hospital setting.

A 12-year-old male (drowning at age 4 with severe neurologic impairment, cerebral palsy: gross motor function classification system level 5, severe scoliosis, exclusively fed via gastrostomy tube and history of recurrent pneumonias over the past year) was admitted from home hospice three days ago for multilobar pneumonia, respiratory distress and increasing secretions. The family brought his Physician Orders for Life-Sustaining Treatment (POLST) form indicating Allow Natural Death (AND). The most recent chest x-ray revealed a "white-out" of both lung fields and is not responding to broad-spectrum antibiotics. You are called to assess the patient because the IV is out, he "doesn't look good" and a dose of antibiotics is due. You go to see the patient.

Background (for narrator reference only, not read aloud)

A 12-year-old male (drowning at age 4 with severe neurologic impairment, cerebral palsy: gross motor function classification system level 5, severe scoliosis, exclusively fed via gastrostomy tube and recurrent pneumonias over the past year) was admitted from home hospice three days ago for multilobar pneumonia, respiratory distress and increasing secretions. The patient has had several hospital visits in the past few months for pneumonia. At the last admission, the family and his physicians changed the patient's resuscitation preference from "full code" to Allow Natural Death (AND) and completed a Physician Orders for Life-Sustaining Treatment (POLST) form.

This admission, the patient has a multi-lobar pneumonia that is not responding to broad-spectrum antibiotics and continues to have severe respiratory distress, increased secretions, no urine output, is pale and requires 10 liters per minute supplemental oxygen via non-rebreather face mask (NRB). His oxygen saturations remain below 80%. The patient appears uncomfortable and is making grunting/moaning sounds. He is dying. The parent(s) is(are) present, worried, and asking the physicians what they can do for him.

NOTES FOR SCENARIO:

Code Status

Allow Natural Death (AND)

Home medication

Glycopyrrolate
Lorazepam
Docusate
Baclofen pump (no changes recently)
Levetiracetam, Phenobarbital

IV Access

Peripheral IV (now out)

Physical Exam

Learner	Narrator Response
**If**	
Asks for vitals	Wt 35kg T 35 HR 65 R 8 BP 65/30 O2 last read 80%, now not registering 10 liters per minute NRB. No urine output for 24 hours
Asks for physical exam	Gen: Dusky, moaning, grunting, clammy
	Resp: shallow breaths; periods of apnea; crackles throughout lung fields, minimal breath sounds. Copious oral secretions and gurgling noises<br>CV: Irregular heart rate, thready pulse<br>Ext: cool, mottled, CR 4-5 sec

Scripted Responses

Parent(s)
Anxious and sad. Parent(s) still has(have) guilt over the drowning that occurred while parent(s) present at a friend's pool party. Parent(s) know that the patient has been deteriorating and is at the end of life, but still find it difficult. The main goal is that the patient be comfortable.

Learner	Parent(s) Response
If	
Suggests Labs	"Will it help?" "He's been poked enough already."
Suggests X-ray	"Will it help?"
Suggests suction	"Won't that just irritate him more?"
Suggests PICU consult	"Why?" "Is he that sick?" "We don't want him to be put on tubes and machines."
Does not address the "death rattle"	"What's that noise?" "He sounds so gurgly." "Is that bad for him?" "Can't you make it go away?"
If provides opiate/anxiolytic	"Thank you, he seems more comfortable, but still has noisy breathing"
If provides anticholinergic	"Thank you, his breathing sounds less noisy"
**Does not explicitly state to the parent(s) that he is dying**	**"Is he dying?"**
Suggests decreasing fluids/feeds	"He needs feeds. Will he die because he's not getting fluids?"
Does not offer psychosocial support (call family, social work, chaplain etc.)	"We feel so alone." "Should we call our family members?"
Does not offer to discontinue monitors	"Can you turn those off?" "I/we can't stop staring at them."
**Does not offer to leave the room after an appropriate amount of time**	**Do you mind if we spend some time alone with our son?**

Endpoint
After the learner has left the room, the narrator fast-forwards the scene 15 min. Parent(s) come(s) out of room and states that "I don't think my(our) child is breathing anymore."

Learner Should	Narrator
Inquire about:<br>1. Breath sounds, Chest Wall Movement<br>2. Pulse<br>3. Pupillary reflex	"All are absent"
**Learner**	**Parent**
Does not offer follow-up plan with or assistance from social work/other resource	"What do we do now?"

Scenario #4: The Dying Child

Date: ___________________________

Resident's Name: ____ Scorer's Name: ____

Role Player's Names: ____

Time Paged: _______ Time Arrived: ________ Time Debriefing Completed: _________

Critical Performance Steps	Descriptions	SCORING<br>**Please circle a score.**<br>1=Did not complete<br>2=Completed with prompting<br>3=Completed with NO prompting
**General**		
Identifies self and role in patient's care		1.................2.................3
Empathetic and effective communication with parent	Places parent and/or patient at ease<br>Uses non-judgmental statements<br>Listens to parent effectively<br>Acts professionally	1.................2.................3
**Patient Management**		
Obtains vitals, performs physical exam	*MUST do both for any credit*	1.................2.................3
Recognizes that the child is dying<br>*Score "1" if does not say form of word "die"*	Explicitly states to the parent that child is dying	1.................2.................3
Recognizes and treats the "death rattle"<br>*MUST offer 4 of 5 for any credit*	Asks if it bothers the parents<br>Explains why it happens/that it's not dangerous<br>Decreases feeds/fluids<br>Repositions<br>Medication: Robinul, Atropine drops, (Scopolomine patch will not reach peak in time but if mentioned, will receive credit)	1.................2.................3
Addresses code status/POLST form		1.................2.................3
Suggests psychosocial support<br>*MUST mention at least 1 for any credit*	Social worker, Child Life, Chaplain, Religious/Spiritual representative from community, Family members, Friends	1.................2.................3
Sets an atmosphere that de-emphasizes medical intervention and fosters comfort and family closeness	Offers to discontinue monitors<br>Turns down lighting<br>Moves machines out of room<br>Music	1.................2.................3
Demonstrates sensitivity to family need for increased/decreased medical presence	Offers to leave room but be available outside room	1.................2.................3
Declare patient's death<br>*MUST address first 2 for any credit*	Assesses breath sounds and pulse<br>Assesses pupils<br>Acknowledges the parents, their courage, love, etc.<br>Calls Attending MD, states time of death	1.................2.................3
**Case Conclusion**		
Is aware of/offers final arrangements and bereavement	Mentions funeral arrangement and bereavement services. Credit even if deferred to social work.	1.................2.................3

Scenario #4: The Dying Child p.2

	Check ONE Only				
	Beginner	Novice	Developing	Near Competent	Competent
	1	2	3	4	5
Confident					
Comfortable					
Empathetic/Sensitive					
Respectful/Professional					
Informative					
Comforting					

Comments:
